# A Dietary Supplement Containing Fucoidan Preserves Endothelial Glycocalyx through ERK/MAPK Signaling and Protects against Damage Induced by CKD Serum

**DOI:** 10.3390/ijms232415520

**Published:** 2022-12-08

**Authors:** Manuel Regier, Carolin Christina Drost, Matthias Rauen, Hermann Pavenstädt, Alexandros Rovas, Philipp Kümpers, Hans Vink, Robert M. Long, Wolfgang A. Linke, Jerzy-Roch Nofer, Alexander-Henrik Lukasz

**Affiliations:** 1 Department of Medicine D, Division of General Internal Medicine, Nephrology, and Rheumatology, University Hospital Münster, 48149 Münster, Germany; 2 Department of Physiology, Cardiovascular Research Institute Maastricht, Maastricht University, 6211 Maastricht, The Netherlands; 3 Microvascular Health Solutions, American Fork, UT 84003, USA; 4 Institute of Physiology II, University Hospital Münster, 48149 Münster, Germany; 5 Center for Laboratory Medicine, University Hospital Münster, 48149 Münster, Germany

**Keywords:** endothelial activation, glycocalyx, fucoidan, ERK/MAPK signaling, endocalyx, chronic kidney disease

## Abstract

(1) Damage to the endothelial glycocalyx (eGC), a protective layer lining the endothelial luminal surface, is associated with chronic kidney disease (CKD), which leads to a worsening of cardiovascular outcomes in these patients. Currently, there are no targeted therapeutic approaches. Whether the dietary supplement Endocalyx^TM^ (ECX) protects against endothelial damage caused by uremic toxins is unknown. (2) We addressed this question by performing atomic force microscopy measurements on living endothelial cells. We examined the effect of ECX on eGC thickness at baseline and with pooled serum from hemodialysis patients. ECX was also successfully administered in vivo in mice, in which eGC was assessed using perfused boundary region measurements by intravital microscopy of cremasteric vessels. (3) Both ECX and fucoidan significantly improved baseline eGC thickness. Our data indicate that these effects are dependent on ERK/MAPK and PI3K signaling. After incubation with eGC damaging serum from dialysis patients, ECX increased eGC height. Intravital microscopy in mice revealed a relevant increase in baseline eGC dimensions after feeding with ECX. (4) We identified a dietary supplement containing glycocalyx substrates and fucoidan as potential mediators of eGC preservation in vitro and in vivo. Our findings suggest that fucoidan may be an essential component responsible for protecting the eGC in acute settings. Moreover, ECX might contribute to both protection and rebuilding of the eGC in the context of CKD.

## 1. Introduction

The endothelial glycocalyx (eGC), a carbohydrate-rich gel-like mesh of large anionic polymers, lines the luminal side of the endothelium along the entire vascular tree [1,2]. It is composed of both hyaluronan and highly sulfated glycosaminoglycans (mainly heparan and chondroitin sulfate) that are attached to core proteoglycans, especially those from the syndecan family [3]. Together with both endothelium- and plasma-derived soluble proteins that are integrated into this mesh, the eGC reaches a thickness of up to 2 µm and thus can be slightly thicker than the endothelial cells themselves [1,4,5]. Its structure in the healthy endothelium is subject to a permanent dynamic equilibrium between the enzymatic or shear-stress dependent removal of or biosynthesis of new eGC components [6]. As it constitutes the interface between blood and endothelium, the intact glycocalyx acts as the primary protective barrier against triggers of vascular diseases including atherosclerosis [7]. Various important physiological properties are attributed to the eGC, such as the regulation of redox state, mediation of shear-induced nitric oxide production or physiologic anticoagulation [1,3,8]. 

Damage to the eGC in vivo has been observed not only in response to inflammatory agents, such as endotoxin [9], TNF-alpha [10], oxidized low-density lipoprotein [8], angiopoietin-2 [11], endothelin-1 [12] and excess atrial natriuretic peptides [13], but also during hypervolemia [14] and hyperglycemia [15]. Many of these eGC-damaging stimuli frequently occur in chronic kidney disease (CKD). Recently, we were able to demonstrate eGC breakdown after exposure to dysfunctional high-density lipoprotein (HDL) isolated from sera of hemodialysis patients [16]. The dysfunctionality of HDL observed in CKD patients is due to uremic toxins, such as symmetric dimethyl arginine (SDMA), serum amyloid A or apolipoprotein C-III, which accumulate in HDL particles and are known to exert endothelial-damaging effects [17,18,19]. These findings are in line with previous data showing both an independent association between eGC damage and renal function impairment and a correlation with endothelial dysfunction in patients with CKD [20,21,22,23].

However, despite the undebatable potential, therapeutic options targeting eGC protection and rebuilding are still lacking [24]. Experimental pharmacological approaches include, among others, the application of spironolactone [25], hydrocortisone [26], albumin [27] or endothelin receptor antagonists [28]. Furthermore, the approach of substituting eGC components is gaining increasing attention [7]. For instance, sulodexide, a synthetic composition of various glycosaminoglycans (GAGs), showed promising results in some, but not all, studies [29,30,31]. Endocalyx^TM^ (ECX) is a dietary supplement composed essentially of antioxidants, GAGs (hyaluronan), glycocalyx substrates (glucosamine sulfate) and fucoidan. Fucoidan is a marine acid polysaccharide consisting mainly of L-fucose and sulfated groups (therefore sometimes referred to as heparan sulfate mimetic [32]) that is known to act as a heparinase inhibitor [33]. Recently, data from experiments with fucoidan showed considerable eGC restoring potential [34]. 

We therefore hypothesized that ECX preserves eGC structure and function, possibly by inhibiting enyzmatic degradation of the eGC and/or by supplying eGC substrates that are incorporated into the eGC. We explored this question using several experimental settings: (i) assessing eGC responses of endothelial-cell monolayers to ECX; (ii) identifying potential eGC-protective signaling pathways activated by ECX; (iii) quantifying the therapeutic effect of ECX in an in vitro CKD model; and (iv) transferring ECX administration and eGC assessment to an in vivo mouse model. 

## 2. Results

### 2.1. ECX Improves the Endothelial Glycocalyx (eGC) Height 

**Figure 1:**
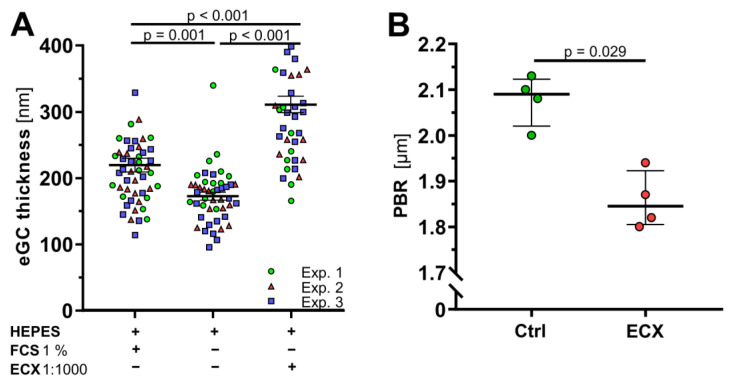
ECX improves endothelial glycocalyx (eGC) thickness in vitro and in vivo. (**A**) Differences in eGC thickness in living endothelial cells (EA.hy926) measured via atomic force microscopy following the addition of 1% fetal calve serum (FCS) or ECX at a dilution of 1:1000 to solvent (HEPES buffer). Each dot represents the mean of 4 to 8 force-distance curves per cell and a minimum of 11 cells, data are presented as mean ± SEM, *n* = 3 independent experiments (Exp.). In all experiments, the incubation time was 60 min. (**B**) Perfused boundary region (PBR)—an inverse parameter of the eGC in vivo—in mice’s cremasteric vessels was obtained with the GlycoCheck^TM^ intravital microscopy system. Mice were fed with either standard diet or ECX-supplemented diet (74 mg/kg) for two weeks before the experiment. Data are presented as median ± IQR, *n* = 4.

As demonstrated before [16], we used atomic force microscopy (AFM) based nanoindentation measurements to identify the effects of ECX on the nanomechanics of the eGC and thus the functional integrity of the eGC in living endothelial cells (EA.hy926). Previous studies revealed that eGC integrity in vitro largely depends on the presence of albumin, or at least 1% fetal calf serum (FCS) [16,27]. After being grown in a culture medium containing 10% FCS and then swapped to test conditions as indicated, the incubation of EA.hy926 cells for 60 min with ECX in an optimal dilution of 1:1000 preserved the eGC height even in the absence of FCS. Both ECX and FCS showed a significantly greater eGC thickness compared to pure HEPES buffer (301.5 ± 11.4 nm vs. 219 ± 9.1 nm vs. 170.9 ± 5.9 nm, *p* < 0.001) (Figure 1A). In dose–response experiments, the 1:1000 dilution showed the most pronounced effect, so we used it for all further in vitro experiments.

The findings were essentially reproducible on primary human umbilical vein endothelial cells (HUVEC) (Supplementary Figure S1).

To further verify these findings in an in vivo mouse model, male *C65BL/6J* mice were fed with either standard diet or ECX-supplemented diet for two weeks. Quantitative intravital microscopy of cremasteric vessels revealed a significant decrease of the perfused boundary region (PBR) in ECX-fed mice, indicating a significant increase of the eGC height in response to ECX (Figure 1B). 

### 2.2. The eGC Thickness Preserving Effect of ECX Is Mediated by Intracellular Signaling Pathways and Is Dependent on Vesicular Transport

**Figure 2:**
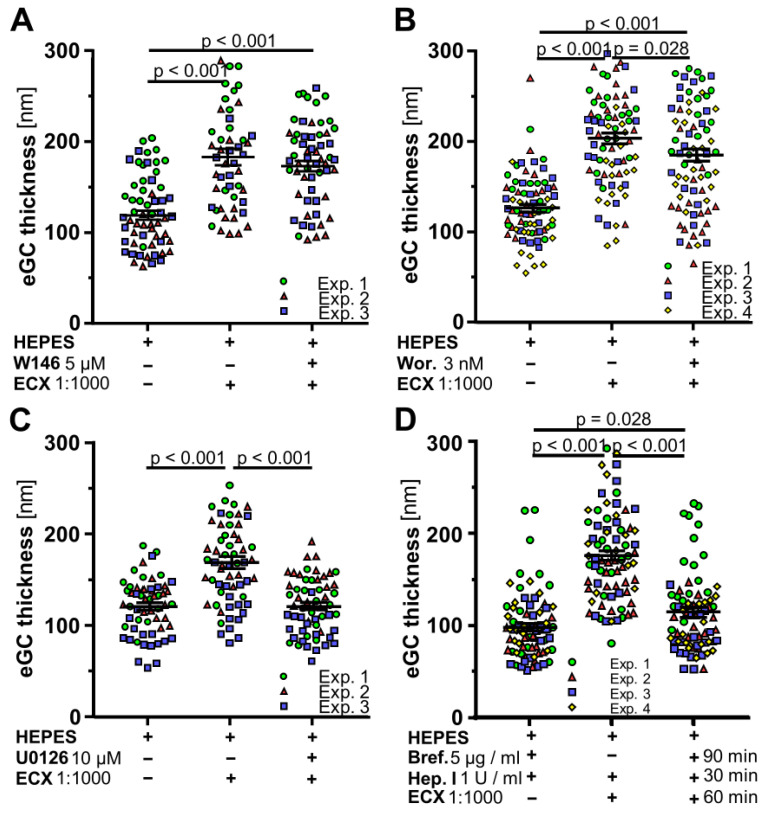
The eGC thickness-preserving effect of ECX is influenced by ERK-MAPK- and PI3K-, but not by S1PR_1_-signaling, whereas the ECX-mediated increase of the eGC thickness is attenuated by inhibition of vesicular transport. (**A**–**C**) Differences in eGC thickness in living endothelial cells (EA.hy926) measured via atomic force microscopy following the incubation with ECX 1:1000 in solvent (HEPES buffer) or the additional incubation with 5 µM of the S1PR_1_ inhibitor W146, 3 nM of the PI3K inhibitor Wortmannin (Wor.) or 10 µM of the ERK-MAPK inhibitor U0126. The incubation time immediately prior to the experiment (Exp.) was 60 min. Pre-incubation with the inhibitors was performed in cell culture medium overnight. (**D**) Here, living endothelial cells were firstly incubated for 90 min with HEPES buffer with or without 5 µM Brefeldin A (Bref.), followed by a 30 min incubation with 1 U/mL heparinase I (Hepar. I) and another 60 min incubation with or without ECX 1:1000 (in solvent still containing Brefeldin A, but not heparinase I), before atomic force measurements were performed. Each dot represents the mean of 4 to 8 force-distance curves per cell and a minimum of 15 cells, data are presented as mean ± SEM, *n* = 3, respectively, 4 independent experiments (Exp.).

The molecular mechanisms of ECX-mediated eGC-protecting effects are unknown. Through explorative literature research we identified several targets potentially responsible for eGC preserving effects [35,36]. We next examined whether or not ECX effects might be mechanistically related to those pathways (Figure 2A–C). Therefore, we used the Sphingosine 1-phosphate receptor (S1PR_1_) antagonist W146 (5 µM) to inhibit its downstream G protein-coupled intracellular pathways. Furthermore, we tested the Phosphoinositide 3-kinases (PI3K) inhibitor Wortmannin (3 nm) and the extracellular signal-regulated kinase (ERK-MAPK) inhibitor U0126 (10 µM). To ensure effective inhibition, the respective inhibitor was pre-incubated 12 h before the experiments. On the next day, AFM measurements were performed after incubation for 60 min in either HEPES buffer with or without ECX (positive or negative control), or HEPES buffer containing ECX and the respective inhibitor. Incubation with W146 showed no significant difference in eGC levels compared to the positive control (Figure 2A). Wortmannin (204.2 ± 6.1 nm vs. 185.6 ± 6.9 nm, *p* = 0.028) caused a slight difference, and U0126 (120.4 ± 4.0 nm vs. 168.4 ± 6.8 nm, *p* < 0.001) caused a more distinct, statistically significant reduction in eGC thickness compared to the positive control (Figure 2B,C). Of note, the eGC thickness after incubation with inhibitor (in the absence of ECX) was essentially equivalent to negative control for all three inhibitors. These data suggest an involvement of ERK-MAPK and potentially PI3K signaling pathway, but do not indicate an involvement of S1PR_1_.

To further narrow down possible downstream mechanisms, we performed experiments with Brefeldin A, a known inhibitor of vesicular transport through the Golgi complex and thus of exocytosis [37]. Firstly, EA.hy926 cells were incubated for 90 min with HEPES buffer with or without 5 µM Brefeldin A to block exocytosis. Then, the eGC was enzymatically removed by 1 U/mL heparinase I for 30 min, a substance known for its eGC digesting capacities [38]. After enzymatic digestion, ECX could restore eGC properties within 60 min, but only in the absence of Brefeldin A (176.6 ± 5.6 nm ECX without Brefeldin A vs. 115.5 ± 5.6 nm ECX with Brefeldin A, *p* < 0.001) (Figure 2D). This indicates an important role for exocytosis as a mechanism of action.

Moreover, these data argue against a prominent sealing effect of the hyaluronan contained in ECX and instead for a genuine molecular mechanism. In this respect, the sulfated polysaccharide fucoidan and glucosamine sulfate seemed particularly interesting. AFM measurements revealed that only fucoidan, but not glucosamine, exerted an acute effect on eGC thickness (Supplementary Figure S2). 

### 2.3. ECX Prevents Enzymatic Degradation of the Endothelial Glycocalyx (eGC) and Protects the eGC from Damage by Uremic Serum 

**Figure 3:**
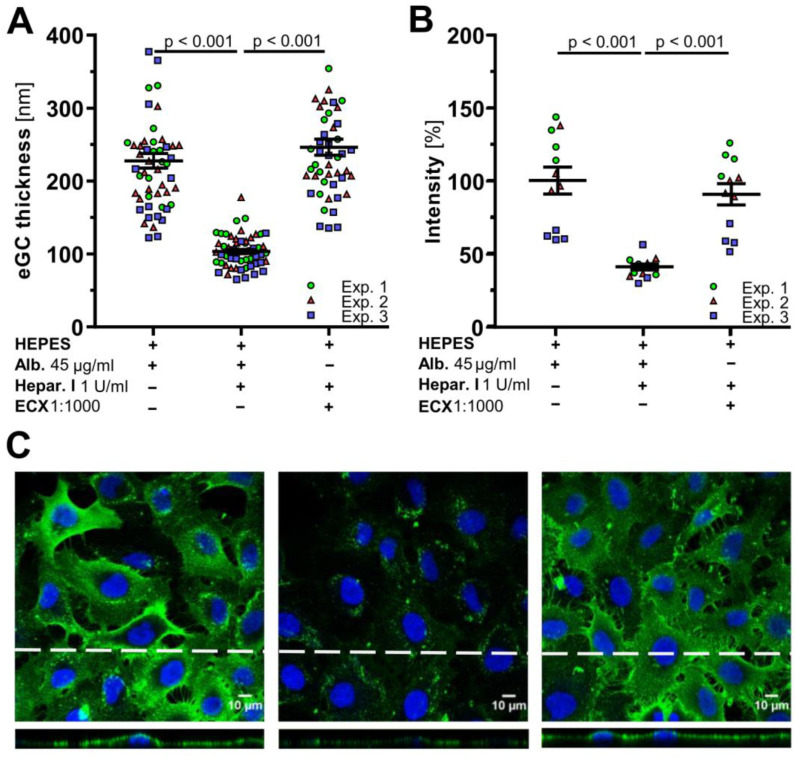
ECX prevents heparinase I induced damage to the endothelial glycocalyx (eGC). (**A**) Differences in eGC thickness in living endothelial cells (EA.hy926) measured via atomic force microscopy following the addition of 45 µg/mL albumin (Alb.), 1 U/mL heparinase I (Hepar. I) and/or ECX 1:1000 to solvent (HEPES buffer). Each dot represents the mean of 4 to 8 force-distance curves per cell and a minimum of 15 cells, data are presented as mean ± SEM, *n* = 3 independent experiments (Exp.). (**B**,**C**) Representative immunofluorescence images and fluorescence intensity analyses of heparan sulfate staining after incubation with albumin (45 µg/mL), heparinase I (1 U/mL) and/or ECX 1:1000), *n* = 3. Data are presented as mean ± SEM percentage compared to control (**C** left picture). (Top) Z-projection and (bottom) cross-sectional images of stack along the dashed lines. Bar = 10 µm. blue = DAPI (4′,6-diamidino-2-phenylindole), green = heparan sulfate. In all experiments (Exp.), the incubation time was 60 min.

Having shown ECX’s ability to regenerate the eGC thickness after enzymatic degradation, we next investigated whether ECX is also able to prevent eGC degradation in vitro when co-administered with heparinase I. AFM experiments on EA.hy926 cells revealed a significantly greater eGC thickness in case of ECX supplementation despite the presence of heparinase I (246.1 ± 11.1 nm vs. 103.6 ± 3.0 nm, *p* < 0.001) (Figure 3A). Confocal microscopy confirmed the abundance of heparan sulfate (HS), a major component of the eGC, in ECX-treated cells, whereas HS was absent on the surface of heparinase-treated cells (Figure 3B,C). 

**Figure 4:**
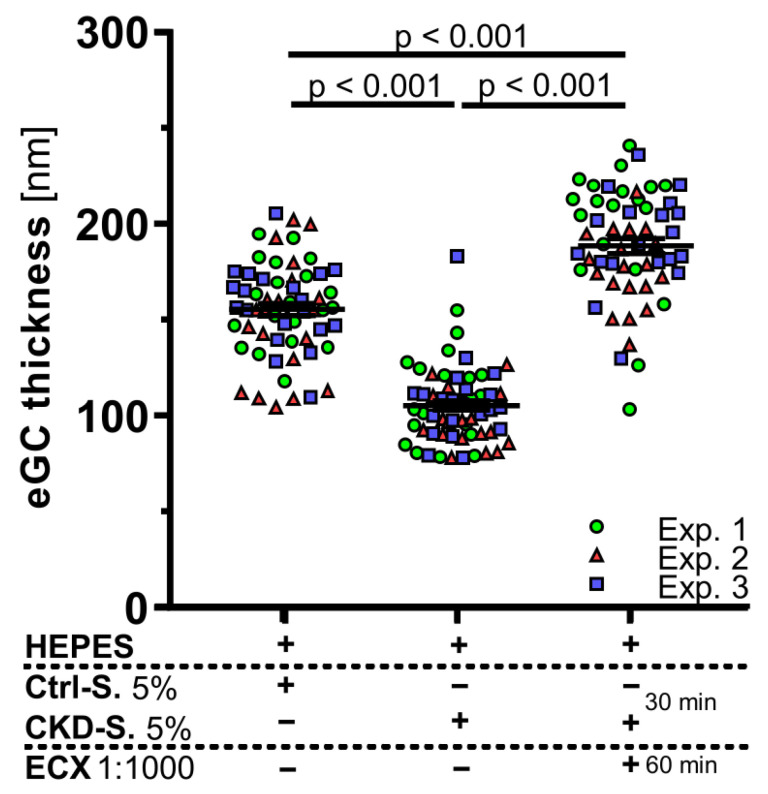
Endothelial Glycocalyx (eGC) damage induced by serum from hemodialysis patients is abolished by ECX in vitro. Differences in eGC thickness in living endothelial cells (EA.hy926) measured via atomic force microscopy following an incubation sequence. Firstly, cells were incubated for 30 min with either 5% serum pooled from patients on hemodialysis (CKD-S.) or 5% serum from healthy controls (Ctrl-S.). Secondly, cells were washed and incubated for 60 min with solvent (HEPES buffer), with or without ECX 1:1000. Each dot represents the mean of 4 to 8 force-distance curves per cell and a minimum of 15 cells, data are presented as mean ± SEM, *n* = 3 independent experiments (Exp.).

Next, we exposed living endothelial cells to pooled sterile-filtered sera derived from patients on hemodialysis (5%, diluted in buffer) or healthy donors (for patient characteristics see Supplementary Table S1). After 30 min of incubation, cells were washed and incubated for 60 min with HEPES buffer with or without ECX. CKD serum led to a sustained decrease in eGC thickness compared to serum from healthy controls (105 ± 2.6 nm vs. 155.4 ± 3.2 nm, *p* < 0.001). This decrease was completely reversed by therapeutic incubation with ECX (188.6 ± 3.8 nm, *p* < 0.001) (Figure 4). 

## 3. Discussion

In the present study, we identified a dietary supplement containing glycocalyx components and fucoidan as a potential therapeutic for eGC preservation in vitro and in vivo. Our experiments suggest that cellular pathways, including PI3K, ERK-MAPK and vesicular transport via the Golgi system, as well as the inhibition of heparanase, are involved in the protective effect, and that fucoidan may be an essential component which protects the eGC in acute settings. ECX seems to be able to mediate both protection and rebuilding of the eGC in the context of enzymatic and CKD-induced eGC damage. Our data further suggest that ECX supplementation may even increase baseline eGC height in a murine model.

To investigate eGC protective effects of ECX, we used several established eGC degradation models including plasma protein depletion (solvent without additional FCS or albumin) [16,27] and enzymatic degradation by heparinase. Methodologically, we determined eGC integrity using AFM-based nanoindentation measurements and immunofluorescence, established in our group for many years [11,21,38]. Furthermore, we performed perfused boundary region (PBR) measurements, an inverse parameter of eGC thickness [39], in an in vivo mouse model via sidestream darkfield microscopy. Originally developed for sublingual intravital microscopy in humans, we adopted the method to analyze PBR in mouse cremasteric vessels [16]. Of note, recent data obtained shortly after the injection of eGC degrading enzymes were in very good agreement with measurements on corresponding ex vivo mouse aortae analyzed with AFM [16]. Although eGC height remains difficult to assess, our findings were consistent and reproducible across methods, suggesting a reliable eGC protective potential of ECX. Several studies investigating the effect of substitution of eGC constituents yielded discrepant results [29,30,40]. However, our findings are in line with a recent trial showing the eGC restoring capacity of fucoidan in the context of COVID-19 induced damage of the eGC [34]. 

Consistent with this result, of the two main components we tested, only fucoidan showed significant eGC protection. Designated as HS mimetic [32], numerous properties with potential for therapeutic application (among others, antithrombotic and anti-inflammatory effects) have been attributed to this compound [41]. Many years ago, fucoidan was described as a heparinase inhibitor in a tumor model [42], and this property has since been demonstrated in several other models [33]. In our experiments ECX was able to reconstitute eGC thickness (as measured by AFM) after enzymatic degradation by heparinase I. Furthermore, it was able to preserve both eGC thickness and fluorescence intensity of HS (as measured by confocal fluorescence microscopy) upon co-incubation with heparinase I. Whether this phenomenon actually underlies a mechanism of action as a heparinase inhibitor cannot be conclusively answered by our methods; however, it seems conceivable.

By showing that the ERK-MAPK- and the PI3K- (but not the S1PR_1_-) inhibitor significantly reduced the eGC thickness upon co-incubation with ECX, we provided evidence for the involvement of these two pathways in mediating the eGC-preserving effects of ECX. It should be noted that the decrease of eGC height caused by Wortmannin was only slightly significant. This led us to suggest a certain, but not dominating, involvement of the PI3K pathway in eGC regulation. Consistent with our findings, Zeng et al. showed that the S1P induced synthesis of eGC is mediated by the PI3K pathway [35]. Similarly, Hara et al. found that activation of p38 MAPK was crucial for the induction of syndecan-4 expression in vascular endothelial cells [36]. Numerous cross-connections and common subpathways of the PI3K and ERK-MAPK pathways exist, e.g., via the Ras molecules [43,44]. The finding that fucoidan enhanced the phosphorylation of, among others, ERK and protein kinase B (AKT) in HUVECs further supports the significance of these two pathways in mediating the fucoidan effects [45]. Moreover, we observed a marked attenuation of the ECX-mediated increase in eGC height by Brefeldin A. This led us to conclude that the (at least short-term) ECX effect may be dependent on exocytosis of eGC components in preformed vesicles rather than supplying constituents that are incorporated into the eGC themselves [37].

Noteworthy is the fact that ECX was able to completely reverse eGC damage triggered by serum of hemodialysis patients. Strong evidence for the pronounced eGC injury of our dialysis patient collective in vivo comes from the fact that both PBR values and serum levels of syndecan-1 were significantly greater than in healthy individuals (Supplementary Table S1). CKD is a major independent risk factor for cardiovascular disease (CVD) [46], while a damaged eGC seems to negatively affect cardiovascular outcomes in CVD patients [47]. A close connection between eGC breakdown, probably an important pathophysiological step in accelerating atherosclerosis (the precursor to CVD [48]), and CKD has been extensively documented [16,20,21,22,23]. Considering the lack of targeted therapy or prevention options, progress in this field is of great importance. In this regard, fucoidan has become of increasing interest in recent years [41]. Our present data suggest that part of its antiatherosclerotic effects might be due to the protection of the eGC. Preserving the eGC might therefore be a relevant factor in maintaining vascular health, especially as this structure has not yet received much attention in this context. It is well known that oxidative stress is a prevalent problem in CKD patients [49] and triggers eGC deterioration [8]. One possible mechanism of eGC protection could be the strong antioxidant activity of fucoidan, which Wang and co-workers have already demonstrated both in vitro and in a CKD rat model in vivo [50,51]. Furthermore, strong upregulation of HS-degrading endothelial heparanase occurs in hemodialysis patients [52] and the heparanase-dependent eGC degradation could be counterveiled by fucoidan, which acts as a heparanase inhibitor [33]. Consistent with our results, a renoprotective effect of fucoidan was recently demonstrated in a murine CKD model [53]. While several clinical ECX trials (e.g., NCT03889236) are ongoing, to date, in vivo data are still sparse, as the bioavailability of this substance is debated. However, a quantitative method to detect fucoidan in human plasma has been described [54]. Moreover, the results of a small trial in aging mice fed with an ECX-supplemented diet for 10 weeks strongly suggested both adequate uptake and efficacy in improving eGC properties [55]. Our preliminary in vivo data consistently demonstrated that an orally administered ECX-supplemented diet in mice could reach and functionally affect systemic circulation. The fact that ECX-fed mice showed a reduced PBR compared to control mice might suggest a “supraphysiological eGC”. In line with this in vivo finding, AFM measurements of endothelial cells incubated with ECX 1:1000 showed a higher eGC thickness than with 1% FCS. However, we cannot exclude the possibility of causing some kind of surgical trauma by performing anesthesia and subsequent cremaster preparation. As our group has previously shown, damage to the eGC is already possible within the very short time span of even five minutes [11]. Although we have trained the method very frequently to limit the damage to a minimum, one might consider a certain “small damage model”, to which the eGC of ECX-fed mice might be more resilient than the eGC of control mice fed with standard diet. To further clarify this, testing ECX in a pathological damage model is certainly very interesting. Clearly, there is also great potential for further translational studies related to CKD, which is why we are currently planning an animal study for the use of ECX in a murine 5/6 nephrectomy model.

One strength of our methodology is the approach of studying the fragile glycocalyx layer on vital endothelium. We performed AFM experiments on living endothelial cells and addressed the translational aspect by conducting PBR measurements with GlycoCheck^TM^ software in vivo. Moreover, we used pooled human serum from dialysis patients with demonstrably elevated uremic toxins, such as SDMA, with potential to damage the eGC in vitro [16]. In our experience, non-invasive sublingual PBR measurements to approximate the eGC height are highly reproducible and accurate, as shown previously [11,16,39]. However, our study has several limitations. First, for most of our experiments, we used an immortalized human hybridoma cell line (EA.hy926), derived by fusing human umbilical vein endothelial cells (HUVECs) with the permanent human lung epithelial cell line A549. Therefore, certain epithelial properties of their eGC cannot be excluded with certainty. However, in many experiments of our group on HUVECs, human pulmonary microvascular endothelial cells (HPMECs) and murine aortic endothelium, the eGC decrease in immunofluorescence and the AFM data were in very good agreement [11,38]. Moreover, we validated this approach in the present work using HUVECs, demonstrating that the ECX effects on eGC height measured by AFM were reproducible. Moreover, the absence of shear stress and/or a lower (non-physiological) amount of plasma protein in the AFM measurements could negatively affect the thickness detection in dense eGC regions that are close to the plasma membrane. An underestimation of the less dense, apical eGC regions seems possible. Additionally, with an ECX incubation time of 60 min in vitro and a mouse feeding time of 2 weeks in vivo, we examined only the short-term effects of this dietary supplement. Therefore, longer-term trials are planned. Furthermore, ECX contains more ingredients than fucoidan and glucosamine, e.g., hyaluronan, antioxidants and enzymes, which may in vivo improve endothelial health by exhibiting beneficial synergistic effects (e.g., protection of existing eGC against damage, synthesis of new eGC components, enhanced eGC repair). It is noteworthy that fucoidan is unlikely to be used as an individual agent alone in clinical practice but instead as a mixed dietary supplement [56,57]. Hence, further experiments are planned to elucidate the role of other ECX components in eGC protection.

## 4. Materials and Methods

### 4.1. Study Population

Sera of haemodialysis patients were derived from a prospective, observational study. The study was carried out in August 2017 in the Kuratorium für Heimdialyse (KFH), a dialysis outpatient clinic cooperating with the University Hospital Münster. The study was performed in accordance with the Declaration of Helsinki and was approved by the local Ethics Committee (Reference: 2016-545-f-S). A total of 30 stable patients on chronic haemodialysis were enrolled in a non-consecutive fashion after obtaining written informed consent. Blood samples were taken from the AV-fistula or dialysis catheter prior to the beginning of the haemodialysis session and before the administration of heparin. Patients were excluded if any of the following factors were present: an active malignancy; acute infection with CRP > 476 nmol/L; age < 18 years and pregnancy. Here, we pooled sera from randomly selected patients.

### 4.2. Atomic Force Microscopy

For determination of the eGC thickness, the AFM nanoindentation technique was used as described previously [11,25,38]. Briefly, cells were analyzed in HEPES-buffer (140 mM NaCl, 5 mM KCl, 1 mM CaCl2, 1 mM MgCl2, 5 mM glucose, 10 mM HEPES) supplemented with or without 1% FCS, respectively, or albumin 45 µg/mL at 37 °C in a fluid chamber with a Nanoscope V Multimode AFM (Veeco, Mannheim, Germany). The addition of slight amounts of plasma protein to the buffer is necessary for eGC preservation in vitro, as the work of our group [16] and others [27] has repeatedly demonstrated. Based on a significant amount of preliminary laboratory work with FCS and albumin, we could conclude that those are comparable in this respect. Incubation time of all AFM experiments was 60 min or as otherwise stated in the figure legend. A triangular cantilever (Novascan Technologies, Boone, NC, USA) with a mounted spherical tip (diameter 10 µm) and a spring constant of 10 pN/nm was used to periodically indent the cells. A laser beam was used to quantify the cantilever deflection. Knowing the force acting on the cantilever, the piezo displacement, and the deflection sensitivity, the thickness of the eGC could be calculated. 

### 4.3. Animals and Sidestream Darkfield Microscopy (GlycoCheck^TM^ System)

Male *C57BL/6J* mice, 8–12 weeks old, were ordered at Charles River Laboratories, Germany, and fed for two weeks with either pelleted standard diet or Endocalyx^TM^-supplemented diet (74 mg/kg Endocalyx^TM^-supplement, Altromin, Germany). For intravital microscopy, mice received 0.9% NaCl (approx. 100 µL) intravenously one hour prior to the measurement, which served as a maintenance fluid to prevent vasoplegia whilst under deep anesthesia. They were anaesthetized with ketamine/xylazine and euthanised immediately after the measurement. All procedures were performed after approval of the local authorities (District Government and District Veterinary Office Münster, Münster, Germany) and conducted in concordance with the guidelines from Directive 2010/63/EU of the European Parliament on the protection of animals used for scientific purposes. 

Intravital microscopy was essentially performed as previously described [16]. Cremasteric microvessels (diameter 5–25 µm) were analyzed with GlycoCheck™ software (Microvascular Health Solutions Inc., Salt Lake City, UT, USA) using the sidestream darkfield (SDF) camera (CapiScope HVCS, KK Technology, Honiton, UK). Briefly, the perfused boundary region (PBR, in µm) serves as an inverse parameter of eGC constitution and is derived from the dedicated software, simplified by analyzing the dynamic lateral movement of erythrocytes (RBC) into the eGC. As a degraded eGC allows more RBCs to indent deeper towards the endothelial cell surface—resulting in increased lateral RBC movement—impaired eGC goes along with increased PBR (Supplementary Figure S3). Further technical information and validation was provided previously [16,39].

### 4.4. Antibodies and Reagents

Mouse monoclonal anti-heparan sulfate (HS) antibody (10E4 epitope, AMS Biotechnology, Abingdon, UK) was used as a primary antibody, and polyclonal goat anti-mouse antibody Alexa Fluor 488 (Thermo Fisher Scientific, Waltham, MA, USA) as a secondary antibody for immunofluorescence staining. Heparinase 1, fucoidan, glucosamine sulfate, Brefeldin A and Wortmannin were ordered from Sigma-Aldrich (Munich, Germany). U0126 was purchased from Cell Signalling Technology Inc., Danver, MA, USA. W146 was purchased from Cayman Chemical, Ann Arbor, MI, USA. ECX was kindly provided by Microvascular Health Solutions, Salt Lake City, UT, USA, and was composed as follows (per capsule, in total 750.75 mg): Fucoidan (85%) 106.25 mg, antioxidants (SOD, catalase, polyphenols) 120 mg, glucosamine sulfate 375 mg, hyaluronic acid (1800–3000 kDa) 17.5 mg, microcrystalline cellulose 130 mg, silicon dioxide 2 mg. ECX was dissolved in DMSO, centrifuged, and the supernatant was sterile filtered and diluted with HEPES buffer as indicated in the results and figure legends.

Concentrations and incubation times of heparinase I, albumin, W146 (we used 5 µM, half the dose specified in the literature, since the cells became detached from the bottom of the dish in a 10 µM solution), Wortmannin, U0126 and Brefeldin A were adopted from previous studies [27,35,38,58,59,60]. 

### 4.5. Confocal Fluorescence Immunocytochemistry

Briefly, cells were fixed for 30 min at room temperature (RT) with 2% paraformaldehyde (PFA) and 0.1% glutaraldehyde. After washing and blocking with 10% normal goat serum for 30 min at RT, samples were incubated overnight with the primary antibody (1:100) at 4 °C. After washing, cells were incubated with secondary antibody (1:300) and DAPI (1:50,000) for 1 h at RT. The mounting medium hardened overnight at 4 °C and the cells were imaged with a Leica DMI 6000B-CS/TCS SP8 laser confocal microscope (objective: HC PL APO CS2 63x/1.40 oil, Leica, Wetzlar, Germany). Image stacks with a size of 0.3 µm were analyzed with ImageJ software (version 1.51p 22, National Institute of Health), as previously described [11].

### 4.6. Cell Culture

The human umbilical vein endothelial cell line EA.hy926 (kindly provided by Cora-Jean Edgell, University of North Carolina, Chapel Hill, USA, who established this cell line) was grown in Dulbecco’s modified Eagle’s medium (DMEM; Invitrogen, Karlsruhe, Germany) supplemented with 1% Pen/Strep and 10% FCS, as previously described [38]. The EA.hy926 cell line is a hybrid cell line derived by the fusion of human umbilical vein endothelial cell line and the human lung carcinoma epithelial cell line A549. Human umbilical vein endothelial cells were grown in Gibco^®^ Medium-199 (Thermo Fisher Scientific, Waltham, MA, USA) supplemented with 1% Pen/Strep, 10% heat-inactivated FCS (30 min at 56 °C), 1% Gibco^®^ LVES (50x) and 1% heparin. 

### 4.7. Statistical Analysis

Data are presented as absolute values with means and standard error of the mean (SEM) or median with interquartile range (IQR). Differences between two groups were tested with Mann–Whitney U test. Differences between ≥ 3 groups were analyzed using the one-way analysis of variance (ANOVA), with Tukey correction for multiple comparisons. To test for differences in the glycocalyx thickness between groups, a nested ANOVA was performed to account for both the number of observations from a single experiment and the number of experiments. Tukey correction was used to control the family wise error rate in the situation of multiple comparisons. All tests were two-sided and significance was accepted at *p* < 0.05. GraphPad Prism Version 9 (GraphPad Prism Software Inc, San Diego, CA, USA) and SPSS 20 (IBM, Armonk, NY, USA) were used for data analysis and figure preparation. 

## 5. Conclusions

In conclusion, we identified a dietary supplement containing fucoidan, among others, capable of mediating eGC preservation and rebuilding in the context of enzymatic degradation and CKD. Furthermore, we provide a solid basis for the translation of these findings into a murine model.

## Supplementary Materials

The following supporting information can be downloaded at: https://www.mdpi.com/article/10.3390/ijms232415520/s1.
