# Supplementary material for: A Dietary Supplement Containing Fucoidan Preserves Endothelial Glycocalyx through ERK/MAPK Signaling and Protects against Damage Induced by CKD Serum
Source: Int J Mol Sci. 2022 Dec 8;23(24):15520. doi: 10.3390/ijms232415520 (PMC9779516; doi:10.3390/ijms232415520)
Supplement: Supplementary file 1 [file ijms-23-15520-s001.zip › ijms-2031353-supplementary.pdf]

Supplementary Figures and Tables

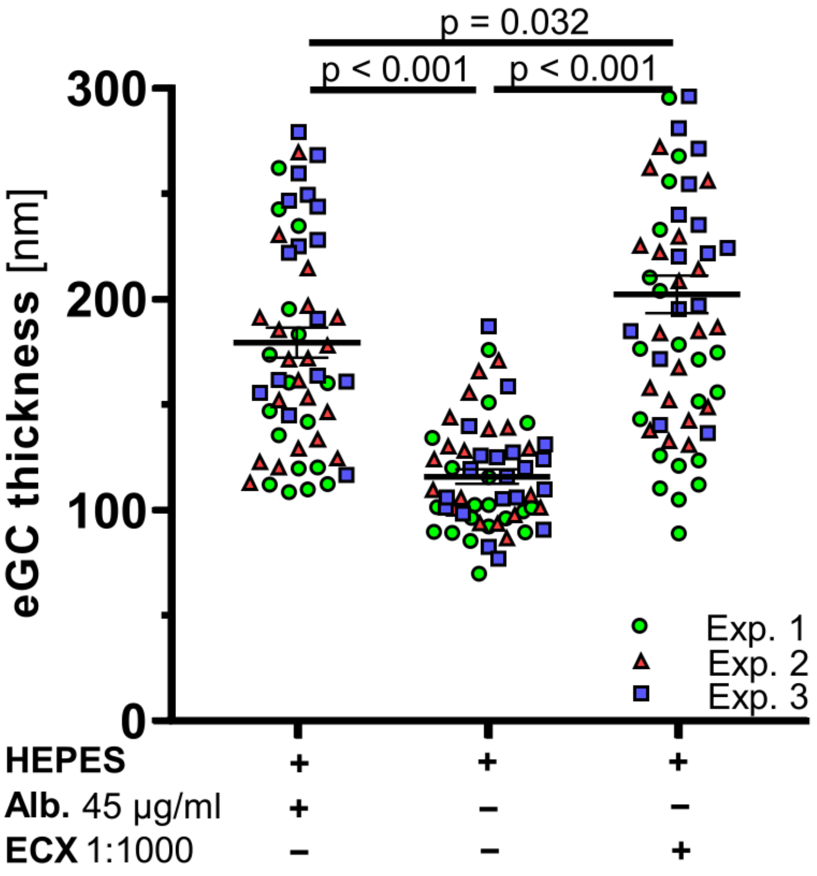

Supplementary Figure S1: ECX preserves height of the endothelial glyocalyx (eGC) in human umbilical vein endothelial cells (HUVECs). Differences in eGC thickness in HUVECs measured via atomic force microscopy following the addition of 45 µg/ml albumin (Alb., corresponding to the mean physiological reference range in human serum) or ECX at a dilution of 1:1000 to solvent (HEPES buffer). Each dot represents the mean of 4 to 8 force-distance curves per cell and a minimum of 15 cells, data are presented as mean ± SEM, *n* = 3 independent experiments. In all experiments (Exp.), the incubation time was 60 minutes.

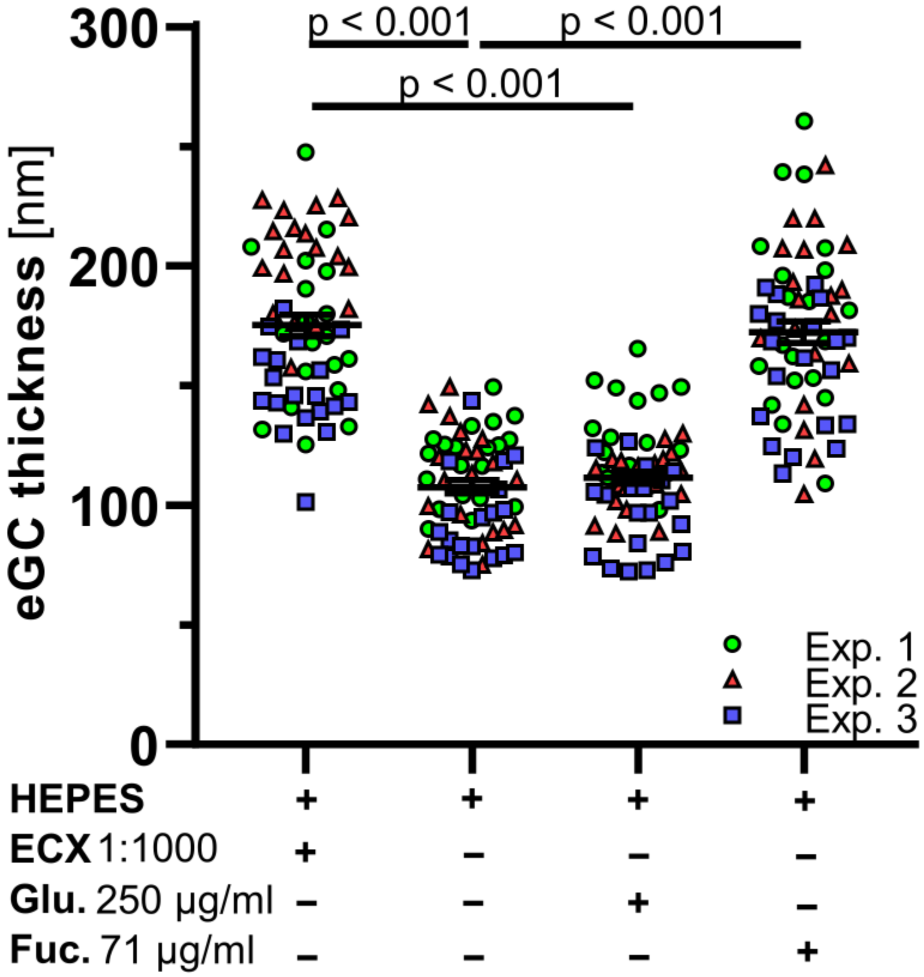

Supplementary Figure S2: The ECX component fucoidan preserves height of the endothelial glycocalyx (eGC) whereas glucosamine sulfate shows no effect**.** Differences in eGC thickness in living endothelial cells (EA.hy926) measured via atomic force microscopy following the addition of ECX at a dilution of 1:1000, 250 µg/ml glucosamine sulfate (Glu.) or 71 µg/ml fucoidan (Fuc.) to solvent (HEPES buffer). Each dot represents the mean of 4 to 8 force-distance curves per cell and a minimum of 15 cells, data are presented as mean ± SEM, n = 3 independent experiments (Exp.). In all experiments, the incubation time was 60 minutes.

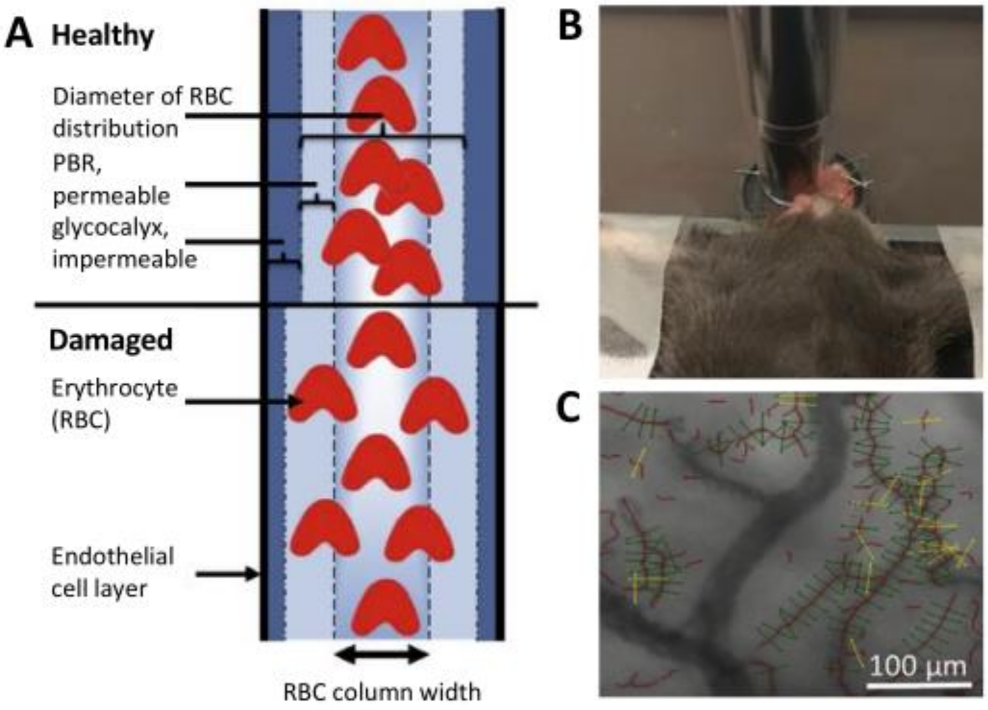

Supplementary Figure S3: *In vivo* estimation of eGC thickness via GlycoCheck^{TM} system in mouse cremaster, modified after Hesse et al. [1] **(A)** Schematic presentation of the perfused boundary region (PBR, in µm) derived from intravital microscopy as indirect parameter of the endothelial glycocalyx. The PBR is calculated by analyzing the dynamic lateral movement of erythrocytes (RBC) into the permeable part of the eGC. In healthy conditions the eGC keeps RBCs distant from the endothelial surface, resulting in low PBR values (upper part of the schematic). As a degraded eGC allows more RBCs to indent deeper towards the endothelial cell surface - resulting in increased lateral RBC movement – impaired eGC goes along with increased PBR (lower part of the schematic) **(B)** Image of the experimental set-up for intravital microscopy with sidestream darkfield camera (GlycoCheck^{TM} software) on prepared mouse cremaster. **(C)** Representative image of microcirculation obtained with the GlycoCheck^{TM} system. The software discards invalid segments (red/yellow lines) and identifies valid vascular segments for further evaluation.

Supplementary Table S1: Demographic, clinical, and laboratory characteristics of patients, modified after Hesse et al. [1]

	Healthy Controls	Hemodialysis Patients	*P value*
Number of patients (n; %)	10 (100)	30 (100)	-
Female sex (n; %)	5 (50)	17 (56.67)	0.71
Age (years, median (IQR))	58.5 (37.5 – 69.25)	68.5 (57.5 – 77.25)	0.06
BMI (kg/m^2, median (IQR))	24.3 (22.98-25.53)	25.64 (20.43 – 29.94)	0.27
Days on dialysis (n; %)	-	1544 (622.3 – 2654.8)	-
Diabetes Mellitus (n; %)	-	8 (26.67)	-
MAP (n; %)	-	91.17 (80 – 105.4)	-
**Causes of chronic kidney insufficiency** (n; %)			-
Vasculitis	-	4 (13)	-
Glomerulonephritis	-	10 (33)	-
Polycystic kidney disease	-	4 (13)	-
Diabetes/hypertension	-	5 (17)	-
Amyloidosis	-	2 (7)	-
Other	-	3 (10)	-
Unknown	-	2 (7)	-
**Hemodynamic data** (median (IQR))			
PBR	1.94 (1.84 – 2.03)	2.1 (1.97 – 2.23)	**0.007**
**Laboratory data** (median (IQR))			
Albumin (g/dl)	4.63 (4.38 – 4.77)	4.11 (3.69 – 4.24)	**< 0.0001**
C-reactive protein (mg/dl)	0.5 (0.5 – 0.5)	0.5 (0.5 – 1.3)	0.06
Syndecan-1 (ng/ml)	28.46 (23.22 – 87.83)	153.6 (97.24 – 282.56)	**< 0.0001**
SDMA (µM)	0.94 (0.87 – 0.97)	2.61 (2.33 – 2.82)	**< 0.0001**
MMP-9 (ng/ml)	611 (343 – 930)	872 (679-1148)	**0.04**

BMI, body mass index; CRP, C-reactive protein; MAP, mean arterial pressure; MMP-9, matrix-metalloprotease-9; PBR, perfused boundary region; RBC, red blood cell; SDMA, symmetric dimethylarginine; Data are presented as n (%) or median (interquartile range). The percentages shown are related to the total number of controls or patients.

Supplementary References

1. Hesse, B.; Rovas, A.; Buscher, K.; Kusche-Vihrog, K.; Brand, M.; Di Marco, G.S.; Kielstein, J.T.; Pavenstädt, H.; Linke, W.A.; Nofer, J.-R.; et al. Symmetric dimethylarginine in dysfunctional high-density lipoprotein mediates endothelial glycocalyx breakdown in chronic kidney disease. *Kidney Int.* **2019**, *97*, 502–515.
